# Potent Antioxidative and UVB Protective Effect of Water Extract of *Eclipta prostrata* L.

**DOI:** 10.1155/2014/759039

**Published:** 2014-02-09

**Authors:** Chin-Feng Chan, Wen-Ying Huang, Hong-Yi Guo, Bo Rong Wang

**Affiliations:** Department of Applied Cosmetology, Hungkuang University, Taichung 43302, Taiwan

## Abstract

Oxidative stress, including Ultraviolet (UV) irradiation-induced skin damage, is involved in numerous diseases. This study demonstrates that water extract of *Eclipta prostrata* L. (WEP) has a potent effect in scavenging 1,1-diphenyl-2-picrylhydrazyl (DPPH), superoxide radicals, and chelating ferrous ion, exhibiting IC_50_ values of 0.23 mg/mL, 0.48 mg/mL, and 1.25 mg/mL, respectively. The WEP total phenol content was 176.45 mg gallic acid equivalents (GAE)/g sample. Chlorogenic acid, a component of the plant's active ingredients, was determined by HPLC and antioxidative assay. However, no caffeic acid, stigmasterol, or wedelolactone was present in WEP. WEP absorbs both UVA and UVB irradiation, and furthermore, the extract shows a dose-dependent response in the protection of HaCaT human keratinocytes and mouse fibroblasts 3T3 cells against UVB-induced cytotoxicity, which may result from a synergistic effect between chlorogenic acid and other active components present in WEP.

## 1. Introduction

The generation of free radicals is a feature of cellular function such as in the mitochondrial respiratory chain, in phagocytosis, and in arachidonic acid metabolism [1]. However, excessive production of free radicals impairs cell membrane integrity [2], causes defects in the susceptible proteins required for mRNA translation, and induces DNA damage and gene mutation [3]. Excess reactive oxidative stress (ROS) which is susceptible to redox dysregulation and oxidative stress is associated with many diseases including atherosclerosis [4], cancer [5, 6], diabetic retinopathy [7], and chronic inflammatory disease [8]. Excess reactive oxygen species is also associated with aging processes [9]. Biological systems evolved endogenous defense mechanisms including employing antioxidants and antioxidative enzymes, to help protect against free radical-induced cell damage [10, 11].

Reactive oxygen species [12] including hydrogen peroxide, superoxide anion, and singlet oxygen are significantly induced in the skin under UV irradiation [13]. Exposure to solar UV radiation is a key factor in the initiation of several skin disorders such as wrinkling, scaling, dryness, pigment abnormalities, and skin cancer [14, 15]. The solar UV spectrum can be divided into three segments based on wavelength bands: UVC: 200–290 nm, UVB: 290–320 nm, and UVA: 320–400 nm. Each spectral range has a characteristic penetration of the epidermal and dermal layers of human skin. Potent antioxidative polyphenols from natural products such as catechins and genistein can prevent UV-induced cytotoxicity [16]. These observations support the notion that UV-induced oxidative stress and damage contribute to melanoma pathogenesis and skin aging and could be targeted using antioxidative preventive therapies. The observations of oxidative stress-induced cytotoxicity that can be attenuated by antioxidative compounds such as alpha-ketoglutarate and N-acetyl cysteine also suggest that oxidative stress plays a pivotal role in the progression of many diseases and could serve as a useful target for treatment [17, 18].

*Eclipta prostrata* L. (Asteraceae) is widely distributed throughout India, China, Taiwan, Thailand, and Brazil. The plant has a folk reputation in Taiwan as a remedy for the treatment of bleeding, hemoptysis and itching, hepatitis, diphtheria, and diarrhea. Stigmasterol, caffeic acid, and wedelolactone have been determined as markers of *E. prostrata* L. Methanol extracts *E. prostrata *L. have been used to treat jaundice, leishmaniasis [19], mouse osteoblast differentiation [20], and hepatic stellate cell proliferation [21]. Water extract of *E. prostrate* L. (WEP) showed a significant reduction in total cholesterol, triglyceride, total protein, and elevation in high-density lipoprotein cholesterol concentrations [22, 23]. The extract is reported to suppress maternal aggression [24]. However, there are no reports of WEP antioxidative activity or the capability of WEP in preventing UV-induced cytotoxicity being investigated or evaluated.

Therefore, the aim of this study is to investigate WEP active ingredients and antioxidative activities. We also review the role of WEP in the prevention of UVB irradiation-induced cytotoxicity.

## 2. Materials and Methods

### 2.1. Materials

2,2-Diphenyl-2-picrylhydrazyl (DPPH; Aldrich), FeCl_2_·4H_2_O (Fluka), trichloroacetic acid (Sigma), phenazine methosulfate (PMS; Sigma), nicotinamide adenine dinucleotide (NADH; Sigma), nitro blue tetrazolium (NBT; Sigma Aldrich), 3-(4,5-dimethylthiazol-2-yl)-2,5-diphenyl tetrazolium bromide (MTT; Sigma), butylated hydroxytoluene (BHT; Aldrich), stigmasterol (Sigma), caffeic acid (Sigma), wedelolactone (Sigma), and ethylenediaminetetraacetate (EDTA; Sigma) were purchased from Sigma Chemical Co. (St. Louis, MO). Chlorogenic acid was purchased from Acros Organics (Thermo Fisher Scientific Inc.). Ferrozine, ferric chloride (FeCl_3_), and potassium ferricyanide (K_3_Fe (CN)_6_) were purchased from Showa Co., Ltd. (Tokyo, Japan). Dulbecco's Modified Eagle's Medium (DMEM; Invitrogen), fetal bovine serum (FBS, Gibco), and penicillin-streptomycin were purchased from Gibco BRL (Life technology, Paisley, Scotland).

### 2.2. Sample Preparation

*E. prostrata *L. was purchased from a traditional Chinese medicine market in Taipei, Taiwan. Fresh aerial parts of the plant were washed, air-dried, weighed, and ground to small pieces. The samples were then immersed in double distilled water (sample to water ratio 1 : 2 by weight) and boiled at 100°C for 20 min. After boiling, samples were filtered through Whatman No.1 paper with vacuum assistance. The water extracts were then freeze-dried and stored at −20°C. Prior to use, samples were dissolved in double distilled water at a concentration of 10 mg/mL to prepare a stock solution.

### 2.3. WEP RP-HPLC Analysis

The RP-HPLC system included a binary pump and UV detector (Agilent infinity 1200). Analytical RP-HPLC of the WEP extract was performed on 20 *μ*L samples injected into a 5C18-AR-II analytical column (250 × 4.6 mm, 5 *μ*m). The mobile phase was a ternary gradient of solvent A (10 mM KH_2_PO_4_, pH 4.0) and solvent B (acetonitrile/methanol/water = 3/1/1). The gradient flow program was 0 min: 100% A, 0% B; 10 min: 90% A, 10% B; 20 min: 80% A, 20% B; 30 min: 60% A, 40% B, at a flow rate of 0.8 mL min^−1^, and elution was monitored at 320 nm. Gallic acid (1 *μ*g/mL) provided the internal control.

### 2.4. Total Phenolic Content

Total phenolic content was measured based on the redox reaction between Folin-Ciocalteu with phenolics in the sample. The total phenolic content was determined using a previously described method [25]. Briefly, various concentrations (0.1 mg/mL, 0.3 mg/mL, 1 mg/mL, 3 mg/mL, and 5 mg/mL) of 0.3 mL *E. prostrata *L. solutions were mixed with 2.4 mL of distilled water and 0.3 mL Folin-Ciocalteu reagent. Double distilled water was used as vehicle control. Sodium carbonate (20%, 0.6 mL) was added to the reaction mixture and allowed to stand for 30 min. The absorbance at 730 nm was measured and compared to a gallic acid calibration curve and expressed as mg gallic acid equivalents (GAE) per gram of sample.

### 2.5. DPPH Free Radical Scavenging Assay

The principle of DPPH method is based on the reduction of DPPH in the presence of a hydrogen donating antioxidant. The scavenging activity of WEP extracts on DPPH radicals was determined using a previously described method [25]. A total of 50 *μ*L of various concentrations (0.01 mg/mL, 0.1 mg/mL, 0.3 mg/mL, 1 mg/mL, and 3 mg/mL) of WEP or chlorogenic acid were mixed with 150 *μ*L of freshly prepared 1 mM DPPH in ethanol. Double distilled water was used as the vehicle control, and ascorbic acid (0.1 mg/mL, 0.3 mg/mL, 1 mg/mL, and 3 mg/mL) was used as positive control. The mixture was kept in darkness for 30 min. DPPH absorbance was then measured at 517 nm using an ELISA reader (TECAN, Austria). Percent activity was calculated using the following equation:

(1) $$\begin{array}{c} \%\text{Activity}=\left[1-\left(\frac{A_{\text{Sample}}}{A_{\text{Blank}}}\right)\right]\times 100. \end{array}$$

The IC_50_ value, which is the sample concentration required for 50% inhibitory activity, was determined by interpolation. Each test was performed in triplicate.

### 2.6. Reducing Power Assay

The reducing power of the WEP extracts was determined by using a previously described method [26]. The method is based on the principle that substances react with potassium ferricyanide (Fe^3+^) to form potassium ferrocyanide (Fe^2+^). Potassium ferrocyanide then reacts with ferric chloride to form ferric ferrous complex which has an absorption maximum at 700 nm. Briefly, 100 *μ*L of WEP at various concentrations (double distilled water) was mixed with phosphate buffer (100 *μ*L, 2 M, pH 6.6) and K_3_Fe(CN)_6_ (100 *μ*L, 1% w/v). Double distilled water was used as a negative control, and BHT (0.1 mg/mL, 0.3 mg/mL, and 1 mg/mL) was used as a positive control. The mixture was incubated at 50°C for 20 min in a water bath. Trichloroacetic acid (10% w/v; 100 *μ*L) was added and the resulting mixture was centrifuged at 1,050 G for 10 min. The supernatant (100 *μ*L) was combined with distilled water (100 *μ*L) and FeCl_3_ solution (20 *μ*L, 0.1% w/v). The absorbance was then measured at 700 nm using a V630 UV-Vis Spectrophotometer (JASCO Co. LTD, Japan).

### 2.7. Fe^2+^-Chelating Capacity Assay

WEP iron-chelating capacity was determined using a method proposed by Dinis et al. [27]. The principle is based on the formation of ferrozine-Fe^2+^ complex and its disruption in the presence of chelating agents. Briefly, 25 *μ*L aliquots of WEP sample at various concentrations between 0.1 and 1 mg/mL were prepared from stock solution and mixed with 175 *μ*L of methanol, 25 *μ*L of 400 *μ*M FeCl_2_·4H_2_O, and 25 *μ*L of 2 mM ferrozine. The mixture stood for 10 min, and the absorbance was then measured at 562 nm using an ELISA reader. EDTA (0.1 mg/mL, 0.3 mg/mL, 1 mg/mL, 3 mg/mL, 5 mg/mL, and 10 mg/mL) was used as a positive control. This test was conducted in triplicate.

### 2.8. Superoxide Radical Scavenging Assay

WEP superoxide anion-scavenging ability was measured using a previously described method [28]. The phenazine methosulfate nicotinamide adenine dinucleotide (PMS-NADH) system generates superoxide radicals, which reduce NBT to a purple-colored diformazan compound. Briefly, reaction solutions containing various concentrations of WEP (50 *μ*L, 0.1 mg/mL to 1 mg/mL) mixed with PMS (80 *μ*M), NADH (1248 *μ*M), and NBT (200 *μ*M) in phosphate buffer (0.1 M, pH 7.4) were incubated at room temperature for 5 min. Double distilled water was used as a negative control, and quercetin (0.1 mg/mL, 0.3 mg/mL, and 1 mg/mL) was used as a positive control. The color was read at 560 nm against blank samples. The superoxide anion radical scavenging percentage was calculated from the following equation:

(2) $$\begin{array}{c} \text{Scavenging}\;\text{effect}\;\left(\%\right)=\left[1-\left(\frac{A_{\text{Sample}}}{A_{\text{Blank}}}\right)\right]\times 100. \end{array}$$

### 2.9. UV Spectrum of WEP

WEP samples with concentrations of 0.01, 0.03, 0.1, 0.2, and 0.3 mg/mL were investigated in the range 200–400 nm using Ultraviolet-visible (UV-Vis) spectroscopy at room temperature.

### 2.10. Determination of UV-Induced Cytotoxicity-Protective Effect

Human keratinocytes (HaCaT cells) and mouse fibroblast cells (3T3) were cultured at a density of 5 × 10^4^ cells/mL in DMEM medium supplemented with FBS (10% v/v), streptomycin (100 *μ*g/mL), and penicillin (10 U/mL) and kept at 37°C under a 5% CO_2_ humidified atmosphere. To determine the protective effect of WEP against 30 mJ/cm^2^ or 60 mJ/cm^2^ UVB-induced toxicity by UV cross-linking (302 nm, UVP CL-1000), HaCaT cells were seeded in a 96-well plate and treated with various concentrations of WEP (0.1–1 mg/mL) for 24 h. The viabilities of HaCaT and 3T3 cells were determined using MTT assay, a colorimetric assay that measures the reduction of yellow MTT by mitochondrial succinate dehydrogenase to an insoluble, colored (dark purple) formazan product. Briefly, MTT (10 *μ*L, 5 mg/mL) was added to each well and stood for 1 h before removal of the supernatant. The remaining formazone crystals were dissolved in 100 *μ*L DMSO, and the absorbance read at 570 nm with an ELISA reader. Cell viability was expressed as a percentage of surviving cells relative to surviving control cell samples.

### 2.11. Statistical Analysis

Three samples were prepared for each assay. The results were expressed as mean and standard deviations. Data analysis included one-way ANOVA, followed by Duncan's Multiple Range Test (*P* < 0.05), and a correlation test using the SigmaStat 3.5 software program.

## 3. Results

### 3.1. RP-HPLC Analysis of WEP

**Figure 1:**
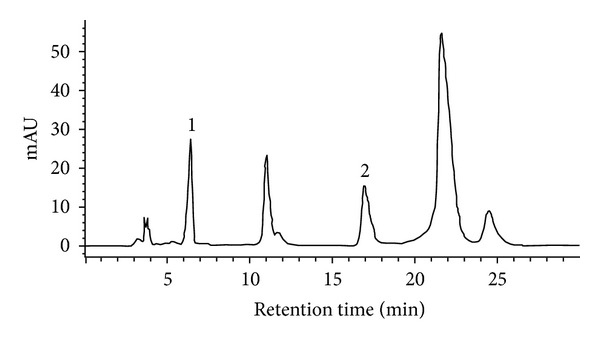
RP-HPLC analyses of water extracts from *E. prostrata *L. Peak 1 is gallic acid used as an international standard. Peak 2 represents chlorogenic acid.

Our determination of compounds present in WEP showed that chlorogenic acid is a major component, present at 1.75 mg/g sample (Figure 1). Wedelolactone, caffeic acid, and stigmasterol, which have been reported as major components of methanol/ethanol extracts of *E. prostrata *L. [12, 21], were not present in WEP. This result indicated that active ingredients of WEP are different from those of methanol/ethanol extracts of *E. prostrata* L., and this may account for their different biological activities [12, 21].

### 3.2. Total Phenolic Content

We measured the total phenolic content of our WEP samples as 175.45 ± 11.56 mg GAE/g (Table 1). Total phenolic content may positively correlate with antioxidative capacity [29]. Therefore, based on our current results, the potent antioxidative effects of WEP may provide profound benefits in combating chronic degenerative disorders, or UV-induced cytotoxicity caused by oxidative stress [19, 30].

### 3.3. DPPH Radical Scavenging Activity

**Figure 2:**
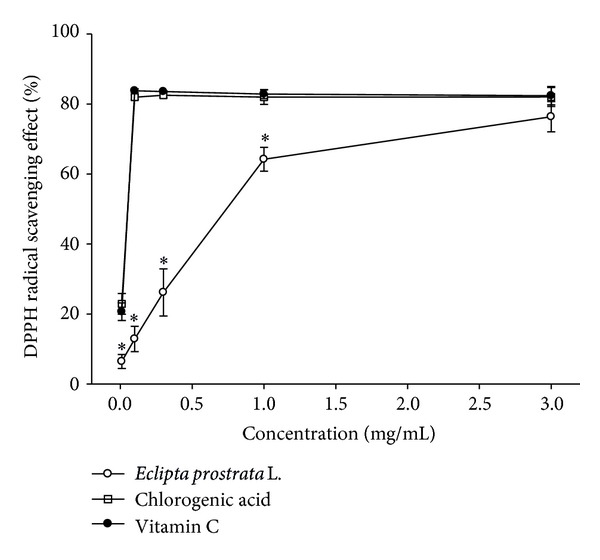
DPPH radical scavenging activity of WEP and chlorogenic acid. Values are means ± SD (*n* = 3). The samples with different lower case letters indicate a significant difference from the vitamin C standard (*P* < 0.05). Vitamin C (0.1 mg/mL, 0.3 mg/mL, 1 mg/mL, and 3 mg/mL) is used as a positive control.

WEP's DPPH radical scavenging activity increased sigmoidally with increasing sample concentrations between 0.01 and 3 mg/mL sample (Figure 2), indicating that WEP's DPPH radical scavenging activity reached a saturation point at concentrations of 3 mg/mL. However, the saturation concentration of chlorogenic acid was 0.1 mg/mL (Figure 1). Chlorogenic acid demonstrated more potent effects than WEP did in scavenging DPPH radicals; the chlorogenic acid and WEP IC_50_ values were 0.050 ± 0.002 mg/mL and 0.75 ± 0.11 mg/mL, respectively [31]. This may explain why DPPH radical scavenging activity observed in our study correlated well with chlorogenic acid content in WEP.

### 3.4. Reducing Power

**Figure 3:**
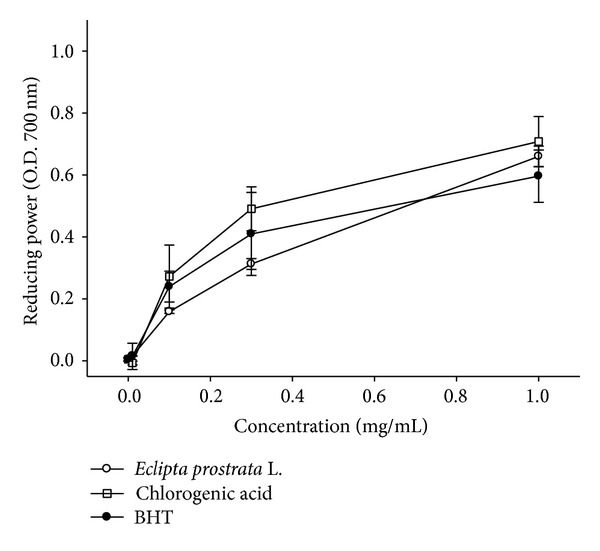
Reducing power of WEP and chlorogenic acid. Values are means ± SD (*n* = 3). Samples with different lower case letters indicate a significant difference from BHT (*P* < 0.05). BHT (0.1 mg/mL, 0.3 mg/mL, and 1 mg/mL) is used as a positive control.

Figure 3 shows a plot of WEP-reducing power as a function of sample concentration. The reduction capacities of chlorogenic acid and BHT were significantly greater than those of WEP (*P* < 0.05) (Figure 3). However, at a concentration of 1 mg/mL, no significant differences in reducing power were observed between WEP, chlorogenic acid, and BHT (Figure 3). Thus, at 1 mg/mL concentration, WEP has a similar efficacy to that of chlorogenic acid and BHT.

### 3.5. Iron-Chelating Capacity

**Figure 4:**
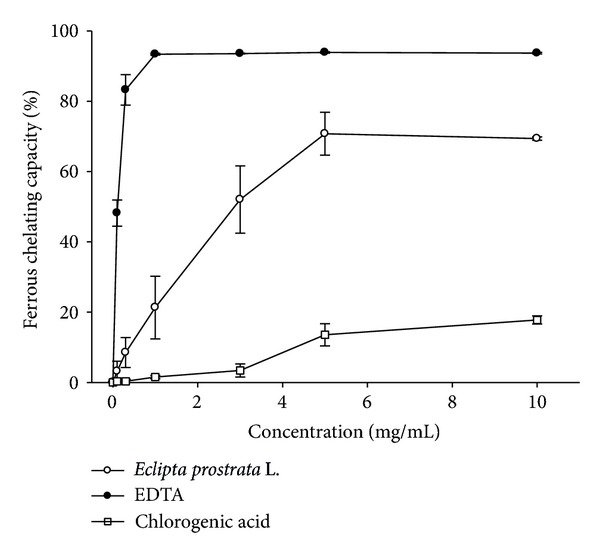
Iron-chelating activity of WEP and chlorogenic acid. Values are means ± SD (*n* = 3). Samples with different lower case letters indicate a significant difference from EDTA (*P* < 0.05). EDTA (0.1 mg/mL, 0.3 mg/mL, 1 mg/mL, 3 mg/mL, 5 mg/mL, and 10 mg/mL) is used as a positive control.

The iron-chelating capacity of WEP samples was measured by assessing their ability to compete with ferrozine in chelating ferrous ion [32]. This test measures activity by the decrease in absorbance of the red Fe^2+^/ferrozine complex.  Figure 4 presents iron-chelating capacity as a function of WEP concentration. The IC_50_ chelating capacity of WEP was 3.20 ± 0.27 mg/mL and reached the saturation point at a concentration of 5 mg/mL. The IC_50_ chelating capacity of chlorogenic acid was 4.5 ± 0.6 mg/mL and reached saturation at 10 mg/mL (Figure 4). This result demonstrates that chlorogenic acid is not a potent iron-chelating agent and makes little or no contribution to WEP iron-chelating activity. The IC_50_ of the EDTA control was 0.110 ± 0.005 mg/mL.

### 3.6. Scavenging of Superoxide Radical Anion

**Figure 5:**
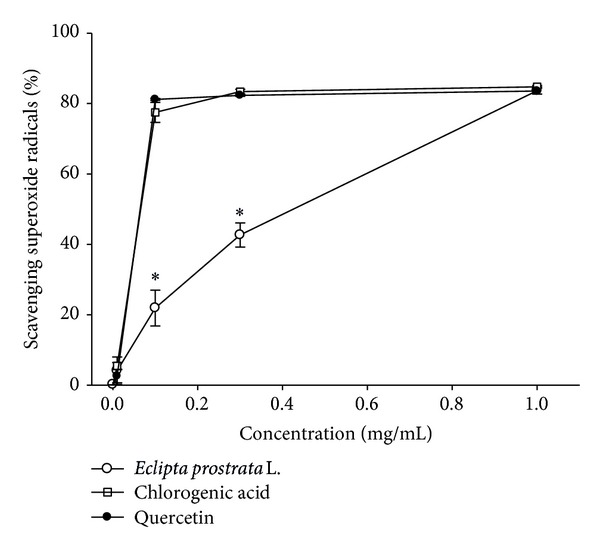
Scavenging activity of superoxide radical of WEP and chlorogenic acid. Values are means ± SD (*n* = 3). Samples with different lower case letters indicate a significant difference from quercetin (*P* < 0.05). Quercetin (0.1 mg/mL, 0.3 mg/mL, and 1 mg/mL) is used as a positive control.

Figure 5 plots the scavenging activity of superoxide radical anion. While a marginal inhibition of 4.10% appeared at a WEP concentration of 0.01 mg/mL, this inhibition increased 83.65% in a dose response manner at WEP concentrations of 1 mg/mL (Figure 5). The WEP IC_50_ for superoxide anion-scavenging activity was 0.48 ± 0.04 mg/mL. The superoxide scavenging activity of chlorogenic acid and quercetin was significantly greater than that of WEP (*P* < 0.05) (Figure 5). However, with 1 mg/mL, we did not observe any significant difference between WEP, chlorogenic acid, and quercetin on superoxide anion-scavenging activity (Figure 5).

### 3.7. UV Spectrum of WEP

**Figure 6:**
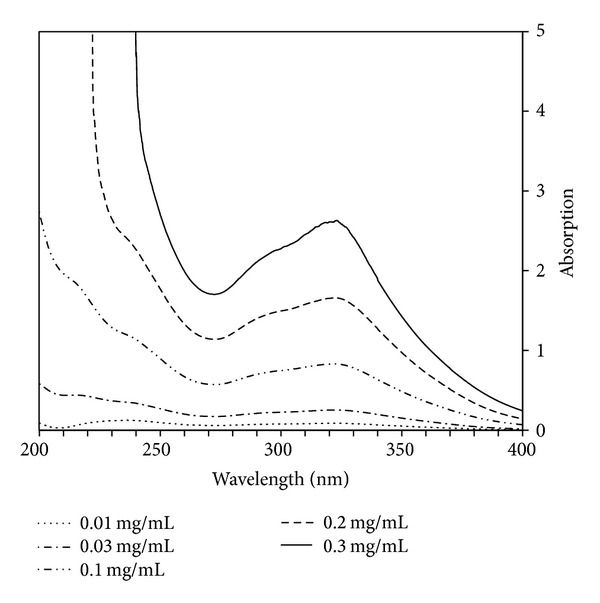
UV spectrum of WEP. The WEP peak absorbance of UVB/UVA occurs at 273 nm in a dose-dependent manner.

WEP exhibits two UV absorbances (Figure 6). One band occurs in the UVC range at 200–275 nm, which is also absorbed by the air. The other band, at 275–400 nm, is classified as UVB/UVA. This absorption range comprises more than 95% of terrestrial UV radiation and is responsible for UV skin damage. WEP peak absorbance of UVB/UVA occurs at 273 nm (Figure 6). The absorbance of WEP at 273 nm is dose-dependent. WEP absorbance at concentrations of 0.01, 0.03, 0.1, 0.2, and 0.3 mg/mL at 273 nm were 0.086, 0.25, 0.83, 1.66, and 2.63, respectively (Figure 6), demonstrating that WEP may serve as an effective UVA and UVB filter.

### 3.8. Protection against UV-Induced Cytotoxicity

**Figure 7:**
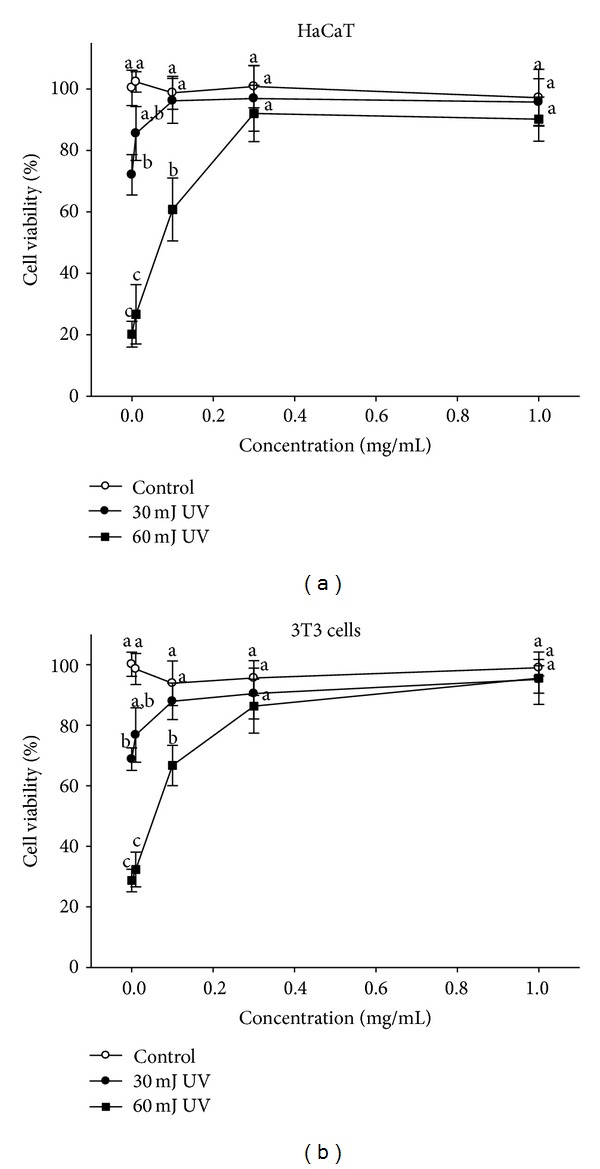
Protective effects of WEP on UVB-induced cytotoxicity. (a) WEP protects against 30 mJ/cm^2^ and 60 mJ/cm^2^ UVB-induced cytotoxicity with a dose-dependent response in HaCaT cells. (b) WEP protects against 30 mJ/cm^2^ and 60 mJ/cm^2^ UVB-induced cytotoxicity with a dose-dependent response in 3T3 cells. Mean values not sharing the same lower case letter are significantly different (*P* < 0.05).

Ultraviolet irradiation induces oxidative stress, resulting in cell damage or cell death [33]. UVB irradiation at 30 mJ/cm^2^ was significantly attenuated by 0.1 mg/mL WEP, and cell viability of HaCaT cells improved from 72.05 ± 6.58%, without WEP protection, to 96.88 ± 10.64%. Furthermore, WEP prevented UVB-induced cytotoxicity from occurring under 60 mJ/cm^2^ irradiation in a dose response manner. At concentrations of 0.3 mg/mL and above, WEP completely prevents UVB-induced cell death (Figure 7(a)). Fibroblast 3T3 cells were more sensitive to UVB-induced cell death compared with HaCaT cells. However, the results were consistent with those of the HaCaT cells; 0.1 mg/mL WEP can significantly attenuate 30 mJ/cm^2^ UVB irradiation and provide significant protection against UVB-induced cell death (Figure 7(b)). Thus, WEP prevents 60 mJ/cm^2^ UVB-induced 3T3 cell death in a dose response manner, and 0.3 mg/mL WEP and greater can almost completely prevent UVB-induced cell death (Figure 7(b)).

## 4. Discussion

Chronic diseases such as neurodegeneration, cardiovascular disorder, diabetes, and cancers have become major health issues in numerous countries and demand considerable healthcare resources. These diseases mainly result from endogenous production of oxidative species. UV radiation is an exogenous ROS-inducing factor and also a critical factor in the initiation and development of a number of skin diseases [34]. According to a WHO report [35, 36], approximately 48,000 melanoma-related deaths occur worldwide per year. A number of antioxidants such as vitamin E and N-acetylcysteine (NAC) exist and are effective in the prevention of reactive oxidative stress- and UV-induced diseases [37]. In recent years, natural product extracts of potent antioxidative activity have arisen, in particular, extracts with high polyphenol contents, such as grape seed proanthocyanidins, resveratrol, silymarin, and genistein, which have demonstrable activity against UV-induced skin inflammation, oxidative stress, and DNA damage [38].

Numerous reports of studies investigate the antioxidative activities of methanol extracts such as those from rosemary or the oil fraction of plants. However, essential oils and methanol extracts of natural products exhibit different biological properties depending on the collection location and reveal seasonal variations [12, 21, 39]. It has been found recently that rosemary constitutes a biomass available for the development of oil-free extracts. The biological activity of oil-free rosemary extract is not sensitive to where the plant was grown [40]. In this study, we used water extract of *Eclipta prostrata* L. which can avoid differences arising from the plant's origin and avoid contamination with organic solvent residues in the final product.

We were unable to find any literature reports that *E. prostrata* L. (Asteraceae) is effective as a remedy for UV-induced skin disease. However, another Asteraceae plant, *Gynura procumbens* Merr., is used as a traditional remedy for various skin diseases in areas of southeast Asia. The ethanolic extract of *Gynura procumbens* inhibits metalloprotease-1 (MMP-1) and metalloprotease-9 (MMP-9) expression induced by UVB irradiation through the inhibition of proinflammatory cytokine mediator release and ROS production [41]. The antioxidative assay results from our study demonstrate that the WEP has potent activity against ROS (Figures 2 to 6) and has rich polyphenol content that includes chlorogenic acid as a major component (Figure 1 and Table 1). WEP protection against UVB-induced cytotoxicity produced similar results in HaCaT and 3T3 cells (Figures 7(a) and 7(b)), indicating that WEP can prevent epidermal (HaCaT) and dermal (3T3) cells against UV-induced cytotoxicity. Chlorogenic acid has been demonstrated as a potent antioxidative polyphenol compound. The scavenging of DPPH radical activity by chlorogenic acid is more effective than that of rutin and ferulic acid, although it is less efficient than caffeic acid and epicatechin gallate [42]. These study results are consistent with previous findings that chlorogenic acid is a potent DPPH radical scavenger, with an IC_50_ of 0.05 ± 0.002 mg/mL (Figure 2). Chlorogenic acid is photostable to UV light and does not degrade under UVA or UVB irradiation [43]. the use of hydrophilic chlorogenic acid with oil in a water-based microemulsion as a vehicle to protect guinea pig dorsal skin against UV-induced oxidative damage [44] has been demonstrated. Chlorogenic acid may play an important role in antioxidative activity and contribute to WEP protection against UVB-induced cytotoxicity [44]. Most of chlorogenic acid's antioxidative activities outperform those of WEP, with the exception of ion-chelating activity (Figure 4). Therefore, we propose that WEP's antioxidative properties and protection against UV-induced cytotoxicity may result from a synergistic effect between chlorogenic acid and other WEP active antioxidants.

## 5. Conclusion

In general, these results suggest that WEP can offer benefits to the pharmaceutical, food, and cosmetics industries to alleviate oxidative and UV-induced skin diseases. However, the detailed mechanism of WEP action against UVB-induced cytotoxicity requires further investigation.

## Figures and Tables

**Table 1:** Total phenolic contents and phenolic compounds of WEP samples.

Sample	Total phenols (mg GAE/g sample)	Chlorogenic acid (mg/mL)	Caffeic acid (mg/mL)
WEP	176.45 ± 11.56	1.75 ± 0.01	ND

ND: not determined.

## Abbreviations

UV: Ultraviolet
WEP: Water extract of *Eclipta prostrata* L.
DPPH: 2,2-Diphenyl-2-picrylhydrazyl
GAE: Gallic acid equivalents
HPLC: High-performance liquid chromatography
HaCaT cells: Human keratinocytes
3T3: Mouse fibroblast cells
ROS: Reactive oxidative stress
PMS: Phenazine methosulfate
NADH: Nicotinamide adenine dinucleotide
NBT: Nitro blue tetrazolium
FeCl_3_: Ferrozine, ferric chloride
K_3_Fe (CN)_6_: Potassium ferricyanide
MTT: 3-(4,5-Dimethylthiazol-2-yl)-2,5-diphenyl tetrazolium bromide
BHT: Butylated hydroxytoluene
EDTA: Ethylenediaminetetraacetate
DMEM: Dulbecco's Modified Eagle's Medium
FBS: Fetal bovine serum
ELISA: The enzyme-linked immunosorbent assay
NAC: N-Acetylcysteine
MMP-1: Metalloprotease-1
MMP-9: Metalloprotease-9
IC_50_: Half maximal inhibitory concentration.
